# Integrated transcriptomic analysis on small yellow follicles reveals that sosondowah ankyrin repeat domain family member A inhibits chicken follicle selection

**DOI:** 10.5713/ajas.20.0404

**Published:** 2020-10-13

**Authors:** Conghao Zhong, Zemin Liu, Xibo Qiao, Li Kang, Yi Sun, Yunliang Jiang

**Affiliations:** 1 Shandong Provincial Key Laboratory of Animal Biotechnology and Disease Control and Prevention, College of Animal Science and Veterinary Medicine, Shandong Agricultural University, Taian 271000, China; 2 Shandong Jihua Poultry Breeding Co. Ltd., Rizhao 276800, China

**Keywords:** Chicken, Follicle Selection, Transcriptome, *SOWAHA*, *Wnt4*

## Abstract

**Objective:**

Follicle selection is an important process in chicken egg laying. Among several small yellow (SY) follicles, the one exhibiting the highest expression of follicle stimulation hormone receptor (*FSHR*) will be selected to become a hierarchal follicle. The role of lncRNA, miRNA and other non-coding RNA in chicken follicle selection is unclear.

**Methods:**

In this study, the whole transcriptome sequencing of SY follicles with different expression levels of *FSHR* in Jining Bairi hens was performed, and the expression of 30 randomly selected mRNAs, lncRNAs and miRNAs was validated by quantitative real-time polymerase chain reaction. Preliminary studies and bioinformatics analysis were performed on the selected mRNA, lncRNA, miRNA and their target genes. The effect of identified gene was examined in the granulosa cells of chicken follicles.

**Results:**

Integrated transcriptomic analysis on chicken SY follicles differing in *FSHR* expression revealed 467 differentially expressed mRNA genes, 134 differentially expressed lncRNA genes and 34 differentially expressed miRNA genes, and sosondowah ankyrin repeat domain family member A (*SOWAHA*) was the common target gene of three miRNAs and one lncRNA. *SOWAHA* was mainly expressed in small white (SW) and SY follicles and was affected by follicle stimulation hormone (FSH) treatment in the granulosa cells. Knockdown of *SOWAHA* inhibited the expression of Wnt family member 4 (*Wnt4*) and steroidogenic acute regulatory protein (*StAR*) in the granulosa cells of prehierarchal follicles, while stimulated *Wnt4* in hierarchal follicles. Overexpression of *SOWAHA* increased the expression of *Wnt4* in the granulosa cells of prehierarchal follicles, decreased that of *StAR* and cytochrome P450 family 11 subfamily A member 1 in the granulosa cells of hierarchal follicles and inhibited the proliferation of granulosa cells.

**Conclusion:**

Integrated analysis of chicken SY follicle transcriptomes identified *SOWAHA* as a network gene that is affected by FSH in granulosa cells of ovarian follicles. *SOWAHA* affected the expression of genes involved in chicken follicle selection and inhibited the proliferation of granulosa cells, suggesting an inhibitory role in chicken follicle selection.

## INTRODUCTION

In regularly laying hens, there are three types of ovarian follicles differing in diameter, including slow-growing follicles (1- to 5-mm), prehierarchal follicles (6- to 8-mm diameter) and preovulatory hierarchy follicles (F6 to F1) [1]. During the process of follicle growth, after the recruitment of primary follicle, only some of prehierarchal follicles, also called small yellow (SY) follicles, will continue developing and finally ovulate. For egg laying, the F1 follicle will ovulate and subsequently one SY follicle with the highest expression of follicle stimulation hormone receptor (*FSHR*) will be selected to become hierarchal follicle [2]. This process is called follicle selection, which is an essential step impacting egg laying performance in chicken.

The SY follicles represent an important developmental stage in chickens, thus attract more attention. By Solexa sequencing, the expression patterns of the five miRNAs were analyzed in different developmental stages in chicken ovaries and in the follicles of various sizes including SY follicles [3]. Analysis on the transcriptomes of chicken SY follicles differing in *FSHR* mRNA expression indicated that Wnt signaling pathway was significantly enriched in the follicles with the greatest fold change in *FSHR* expression [4]. Global gene and protein expression in chicken SY follicles in response to acute heat stress was investigated and 176 genes and 93 distinct proteins with differential expressions were identified, among which the expression of heat shock proteins and peroxiredoxin family were upregulated [5]. Comparing gene expression between SY follicles in chickens and cattle at different follicular development stages revealed a role of estrogen receptor 2 in regulating cytochrome P450 family 19 subfamily A member 1 expression in the theca cells of chicken SY follicles [6]. During follicle selection from SY to F6 follicles, both the ^m6^A methylation peaks and the ^m6^A modified transcripts increased, which lead to the dynamic expression of many folliculogenesis relevant genes, including genes involved in Wnt signaling pathway [7]. Transcriptomic and proteomic comparison between chicken SY and F6 follicles revealed a role of very low density lipoprotein receptor in chicken follicle selection [8].

Some RNA molecules including miRNA [9] and lncRNA [10] play key roles in regulating the expression of protein coding genes. For chicken SY follicles differing in *FSHR* expression, we hypothesize that the differentially expressed genes (DEGs) between different follicles in the same individual might be regulated by miRNA and lncRNA. Therefore, in this study, by integrated transcriptomic analysis on the expression differences in the level of mRNA, miRNAs and lncRNAs in chicken SY follicles, we identified differentially expressed mRNA genes, miRNA and lncRNA genes. Gene interaction network analysis and subsequent functional *in vitro* test revealed that sosondowah ankyrin repeat domain family member A (*SOWAHA*) plays inhibitory role in chicken follicle selection.

## MATERIALS AND METHODS

### Animals and sample collections

Jining Bairi hens (a Shandong Indigenous chicken breed distributed mainly in Jining city) 35 to 40 weeks of age with regular laying for at least one month were used in this study. All sampled 60 Jining Bairi hens were killed by cervical dislocation immediately. Then, the ovaries including all sized follicles were carefully obtained from each chicken, prehierarchal follicles and hierarchal follicles were seperately collected to prepare granulosa and theca cells. The Institutional Animal Care and Use Ethics Committee of Shandong Agricultural University reviewed and approved all procedures described in this study (Permit Number: 2,007,005). This study was performed in accordance with the “Guidelines for Experimental Animals” of the Ministry of Science and Technology of China.

### Library construction and sequencing

Total RNA was extracted from all of the SY follicles (6 to 8 mm) using TRIzol reagent (Invitrogen, Wilmington, DE, USA) and treated with DNase (TaKaRa, Dalian, China) to remove potential genomic DNA contamination, following the manufacturer’s instructions. The quantity and purity of the total RNA were evaluated. The RNA was then divided into two aliquots that were used for library construction of either small RNA or RNA. Fragmented RNA (the average length was approximately 200 bp) were subjected to first strand and second strand cDNA synthesis followed by adaptor ligation and enrichment with a low-cycle according to instructions of NEBNextUltra RNA Library Prep Kit for Illumina (NEB, Ipswich, MA, USA). The purified library products were evaluated using the Agilent 2200 TapeStation and Qubit2.0 (Life Technologies, Carlsbad, CA, USA). The libraries were paired-end sequenced (PE150, Sequencing reads were 150 bp) at Guangzhou RiboBio Co., Ltd. (Guangzhou, China) using IlluminaHiSeq 3000 platform.

### Pre-processing of sequencing reads and quantification of gene expression level

Raw fastq sequences were treated with Trimmomatic tools (v 0.36) using the following options: TRAILING:20, MINLEN: 235 and CROP:235, to remove trailing sequences below a phred quality score of 20 and to achieve uniform sequence lengths for downstream clustering processes. Sequencing read quality was inspected using the FastQC software (v 0.11.6). Adapter removal and read trimming were performed using Trimmomatic (v 0.36). Sequencing reads were trimmed from the end (base quality less than Q20) and filtered by length (less than 25). Paired-end reads were aligned to the chicken reference genome GRCg6a with HISAT2. HTSeq v0.6.0 was used to count the reads numbers mapped to each gene. The whole sample expression levels were presented as RPKM (expected number of Reads Per Kilobase of transcript sequence per Million base pairs sequenced).

### Gene ontology terms and KEGG pathway enrichment analysis of differential expressed genes

The statistically significant DE genes were obtained by an adjusted p-value threshold of <0.05 and |log2 (fold change)| >1 using the DEGseq software. Hierarchal clustering analysis was performed using the R language package gplots according to the RPKM values of differential genes in different groups, and colors represent different clustering information, such as the similar expression pattern in the same group, including similar functions or participating in the same biological process (BP). All differentially expressed mRNAs were selected for gene ontology (GO) and Kyoto encyclopedia of genes and genomes (KEGG) pathway analyses. GO was performed with KOBAS software (v 3.0). GO provides label classification of gene function and gene product attributes (http://www.geneontology.org). GO analysis covers three domains: cellular component (CC), molecular function (MF) and BP. The differentially expressed mRNAs and the enrichment of different pathways were mapped using the KEGG pathways with KOBAS3.0 software (http://www.genome.jp/kegg).

### Co-expression network of differentially expressed lncRNAs and mRNAs

To investigate the potential functions of differentially expressed lncRNAs and the interactions between mRNAs and lncRNAs, we constructed lncRNA/mRNA transcripts co-expression network by calculating the Pearson correlation coefficient and p-value between multiple genes. In this study, the transcripts were filtered using a COR of >0.85 and a p-value of <0.05. Forty-four differentially expressed mRNA transcripts that were enriched in reproduction-related pathways and all differentially expressed lncRNAs were selected to construct a co-expression network that was illustrated using Cytoscape software (v 3.5.1).

### Cell culture

Prehierarchal follicles were treated with 0.1% collagenase II (MP Biomedicals, Santa Ana, CA, USA) at 37°C for 8 min to disperse follicular granulosa cells and for an additional 30 min to disperse follicular theca cells. Theca cell and granulosa cell layers from each hierarchal follicle were collected and combined within their respective group and then dispersed for culture as previously described [4]. Hierarchal follicular granulosa cell layers were dispersed by treated with pancreatin (Gibco, Camarillo, CA, USA) for 15 min, while theca cell layers were dispersed with collagenase II for 30 min. The isolated theca and granulosa cells were planted in a 24-well culture plate containing 1 mL of M199 complete medium with high glucose (Gibco, USA) plus 10% fetal bovine serum (Biological Industries, Kibbutz Beit-Haemek, Israel).

### Overexpression and knockdown assay

The entire coding region of chicken *SOWAHA* gene was amplified and polymerase PrimeSTAR (TaKaRa, China) was used to ensure high fidelity. The polymerase chain reaction (PCR) fragments were generated by double enzyme digestion and ligated with pcDNA3.1(+) expression vectors (Invitrogen, USA) by T4 DNA ligase, which were transformed into DH5α (TransGen Biotcch, Beijing, China) competent cells. After being confirmed by bidirectional sequencing and purified using an EndoFree Plasmid Purification Kit (Qiagen, Valencia, CA, USA), these plasmids were transfected into cells. Empty pcDNA3.1(+) vector was used as the control. For knockdown assay, siRNA was designed according to the chicken *SOWAHA* mRNA sequence (GenBank accession number XM_015294072). The negative control of the siRNA had the same composition with the siRNA sequence but had no homology with *SOWAHA* mRNA (Shanghai Gene-Pharma Co., Shanghai, China). The most effective siRNA was used to analyze the knockdown effect of siRNA on chicken *SOWAHA* gene.

### Cell transfection and follicle stimulation hormone treatment

Granulosa cells were transfected with pcDNA3.1-SOWAHA overexpression plasmid or siRNA when grown to 80% confluency using NanoFectin Transfection Reagent (Shanghai ExCell Biology, Shanghai, China). As for FSH treatment, the cells were cultured with serum-free M199 medium in the absence or presence of different concentration of FSH (Sigma, St. Louis, MO, USA). Twenty-four hours after transfection and FSH treatment, the cells were lysed for RNA extraction.

### Real-time quantitative polymerase chain reaction

The total RNA was extracted from all of the follicles using TRIzol reagent (Invitrogen, USA), and the total RNA from the cultured cells was isolated using a MicroElute Total RNA Kit (Omega, Norcross, GA, USA). The quality of the total RNA samples was tested by gel electrophoresis and spectrophotometry. The cDNA was synthesized using a Primescript RT reagent Kit with gDNA Eraser (TaKaRa, China), and the resultant cDNA was stored at −20°C for mRNA expression analysis. Real-Time quantitative PCR (qRT-PCR) was conducted on an MX3000p instrument (Stratagene, La Jolla, CA, USA) using the SYBR premix ExTaq (TaKaRa, China). Melting curves were used to confirm the specificity of each product, and the PCR efficiencies were determined by analysis of twofold serial dilutions of cDNA that were designed to detect all the signals in the spanning region. The efficiencies were nearly 100%, and therefore, the 2^–^^ΔΔ^^CT^ method for calculating the relative gene expression levels was used [11], and *β-actin* gene was used as the internal control. Primer sequences used for qRT-PCR are shown in Table 1.

### Cell proliferation assay

The proliferation of granulosa cells was detected using an Enhanced Cell Counting Kit-8 Assay Kit (Beyotime, Beijing, China). Approximately 6×10^3^ cells were seeded in every well of a 96-well plate. The cells were transfected with the pcDNA3.1-SOWAHA and empty pcDNA3.1 when the cells reached 60% confluence. At 0, 24, 48, and 72 h after transfection, 100 μL of medium with 10 μL of CCK8 was added to each well, and then the plates were incubated for a further 1 h at 38°C. The absorbance was evaluated using an ELx808 Absorbance Reader at 450 nm.

### Statistical analysis

All data are presented as the mean±standard error of the mean. The differences between groups were determined by one-way analysis of variance followed by Duncan’s test using the SPSS software (SPSS Inc., Chicago, IL, USA). GO and KEGG analyses were assessed by Fisher’s t-test. For statistical analysis, p<0.05 was considered as significantly different.

## RESULTS

### Differentially expressed mRNA, lncRNA and miRNA genes between two small yellow follicles differing in *FSHR* expression in chickens

**Figure 1:**
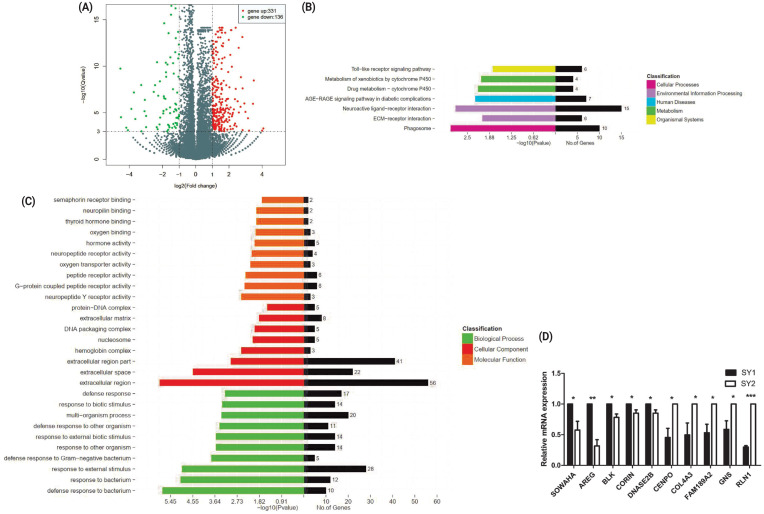
The DEGs between two small yellow follicles differing in *FSHR* expression in chickens. (A) Differentially expressed genes between samples. (B) The KEGG analysis diagram of the DEGs. (C) The GO analysis diagram of the DEGs. (D) Validation by qRT-PCR of 10 DEGs obtained by RNA-seq. SY1 represents small yellow follicles with higher *FSHR* expression. SY2 represents small yellow follicles with lower *FSHR* expression. DEGs, differentially expressed genes; *FSHR*, follicle stimulation hormone receptor; KEGG, Kyoto encyclopedia of genes and genomes; GO, gene ontology; qRT-PCR, real-time quantitative polymerase chain reaction; SY, small yellow.

Previous studies suggest that among the eight to 10 SY follicles in laying hens, the one with highest *FSHR* expression will be selected to become hierarchichal follicle. Therefore, in this study, we compared the transcriptomes between two chicken SY follicles differing in *FSHR* expression. A total of 467 DEGs, including 331 upregulated and 136 downregulated genes were identified between two chicken SY follicles differing in *FSHR* expression at the significant criteria of (|log2 (fold change)| >1 and padj <0.001) (Figure 1A). KEGG analysis showed that these DEGs were mainly enriched in pathways of neuroactive ligand-receptor interaction and cell process-related phagocytosis related to environmental information processing (Figure 1B) and GO analysis showed that most of these DEGs were related to the function of cell composition in BPs (Figure 1C). Quantitative real-time PCR on 10 of these DEGs showed that their expression level was similar to the sequencing data, including *SOWAHA*, amphiregulin, BLK proto-oncogene, Src family tyrosine kinase, corin, serine peptidase, deoxyribonuclease 2 beta, centromere protein O, collagen type IV alpha 3 chain, family with sequence similarity 189 member A2, glucosamine (N-acetyl)-6-sulfatase, and relaxin 1, suggesting that the RNA-seq result was reliable (Figure 1D).

**Figure 2:**
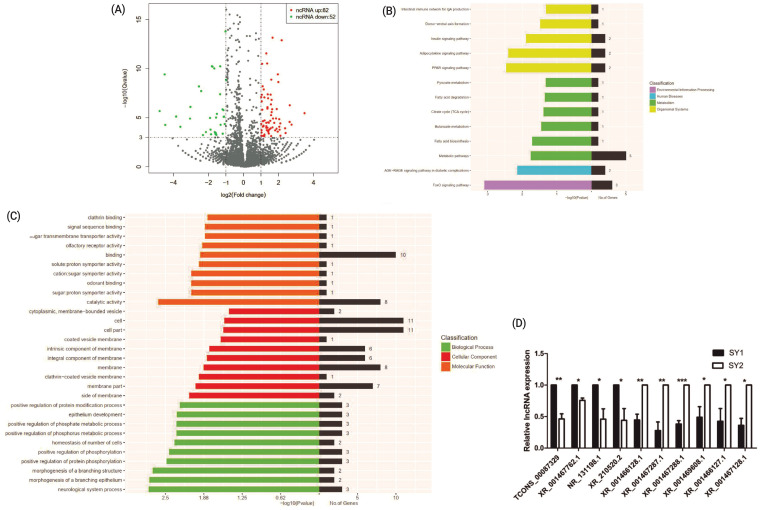
Differentially expressed lncRNA genes between two small yellow follicles differing in *FSHR* expression in chickens. (A) Differentially expressed lncRNAs between samples. (B) The KEGG analysis diagram of the differentially expressed lncRNAs. (C) The GO analysis diagram of the differentially expressed lncRNAs. (D) Validation by qRT-PCR of 10 differentially expressed lncRNA genes obtained by RNA-Seq. *FSHR*, follicle stimulation hormone receptor; KEGG, Kyoto encyclopedia of genes and genomes; GO, gene ontology; qRT-PCR, real-time quantitative polymerase chain reaction.

A total of 134 differentially expressed lncRNA genes, including 82 upregulated and 52 downregulated genes were identified between two chicken SY follicles differing in *FSHR* expression at the significant criteria of (|log2 (fold change)| >1 and padj <0.001) (Figure 2A). KEGG analysis showed that these differentially expressed lncRNA genes were mainly enriched in metabolic pathways and FoxO signaling pathways (Figure 2B) and GO analysis showed that most of these differentially expressed lncRNA genes were enriched in binding, cell and cell part, while more genes were significantly enriched in cell composition related to the function of cell composition in BPs (Figure 2C). Ten of these differentially expressed lncRNA genes (Figure 2D) were validated by qRT-PCR, which were similar to the sequencing data.

**Figure 3:**
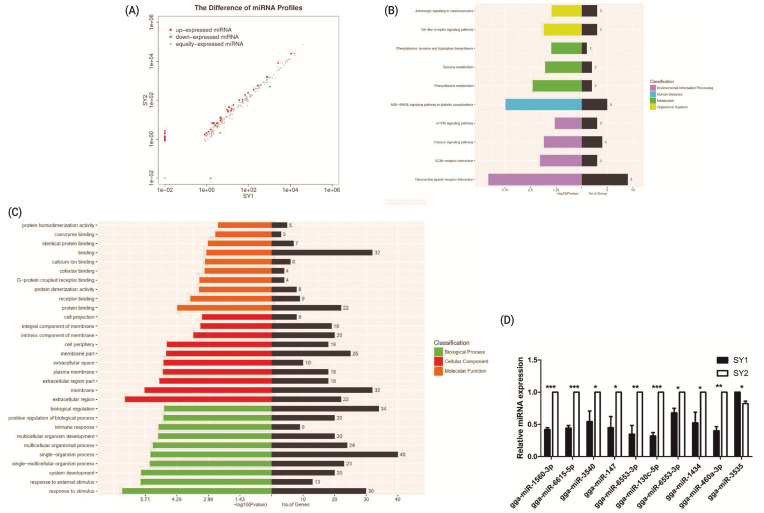
Differentially expressed miRNA genes between two small yellow follicles differing in *FSHR* expression in chickens. (A) Differentially expressed miRNAs between samples. (B) The KEGG analysis diagram of the differentially expressed miRNAs. (C) The GO analysis diagram of the differentially expressed miRNAs. (D) Validation by qRT-PCR of 10 differentially expressed miRNAs obtained by RNA-Seq. *FSHR*, follicle stimulation hormone receptor; KEGG, Kyoto encyclopedia of genes and genomes; GO, gene ontology; qRT-PCR, real-time quantitative polymerase chain reaction.

A total of 34 differentially expressed miRNA genes, including three upregulated and 31 downregulated genes were identified between two chicken SY follicles differing in *FSHR* expression at the significant criteria of (|log2 (fold change)| >1 and padj <0.001) (Figure 3A), and their target genes were predicted. One hundred and forty overlapped genes between the predicted target genes and DEGs were further analyzed by KEGG and GO enrichment analysis, showing that they were mainly enriched in advanced glycation end-recepor for advanced glycation end signaling pathway and neuroactive ligand-receptor interaction pathways (the pathways related to environmental information processing contain the most genes) (Figure 3B), and mostly were related to binding, single organism process, biological regulation, membrane and other significant functions (Figure 3C). Validation by qRT-PCR on 10 of these differentially expressed miRNA genes indicated that the results were consistent with the sequencing data (Figure 3D).

### Integrated analysis on differentially expressed mRNA, lncRNA, and miRNA genes between two chicken SY follicles differing in *FSHR* expression

**Figure 4:**
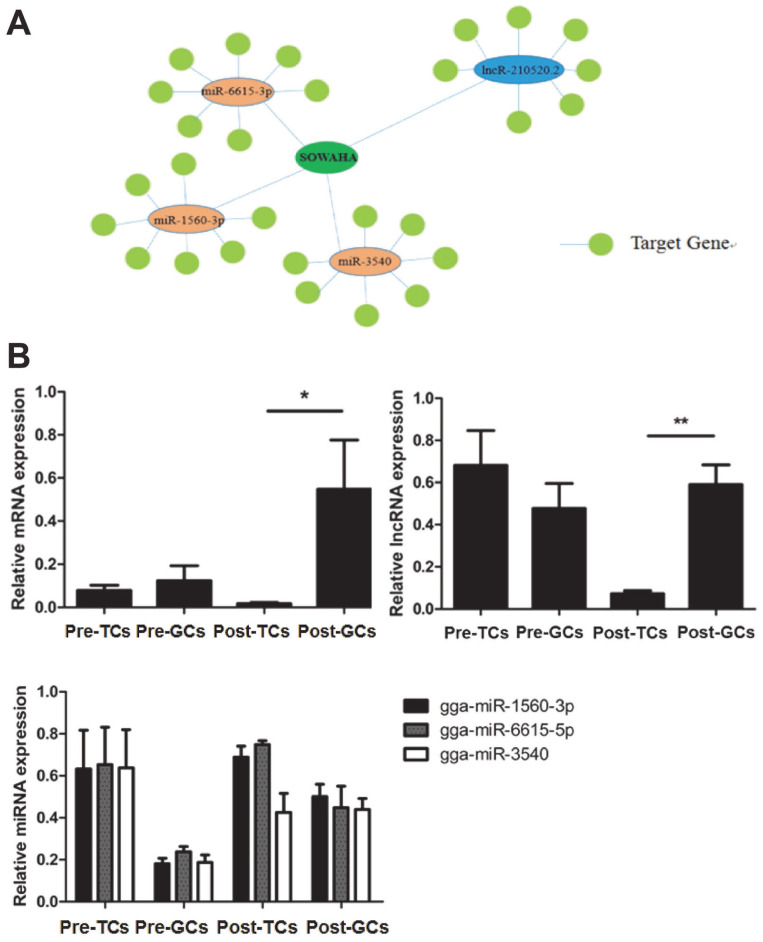
Relationship among *SOWAHA*, *miRNA-6615-3p*, *miRNA-1560-3p*, *miRNA-3540*, and *lncRNA-210520.2* in chicken small yellow follicles (A), prehierarchal and hierarchal follicles (B). Pre-TCs, Pre-GCs, Post-TCs, Post-GCs represent prehierarchal theca cells, prehierarchal granulosa cells, hierarchal theca cells and hierarchal granulosa cells. *SOWAHA*, sosondowah ankyrin repeat domain family member A; TC, theca cells ; GC, granulosa cells.

Integrated analysis indicated that *SOWAHA* is the common target gene of *miRNA-6615-3p*, *miRNA-1560-3p*, *miRNA-3540* and *lncRNA-210520.2* (Figure 4A). Sequencing results showed that, in follicles with higher *FSHR* expression level, the expression of *SOWAHA* and *lncRNA-210520.2* was higher, while that of three miRNA genes was lower. While in follicles with lower *FSHR* expression level, the expression of three miRNA were higher, but that of *SOWAHA* and *lncRNA-210520.2* was lower. The qRT-PCR analysis indicated that the expression of *SOWAHA* in hierarchal follicles was significantly higher in granulosa cells than in theca cells (Figure 4B). The expression of *lncRNA-210520.2* in prehierarchal follicles was different from *SOWAHA*, while in hierarchal follicles, it exhibits similar trend as that of *SOWAHA* (Figure 4C). Changes in the expression of *miRNA-1560-3p*, *miRNA-6615-3p*, and *miRNA-3540* in the granulosa cells and theca cells of hierarchal follicles were contrary to *SOWAHA* (Figure 4D).

### Expression of *SOWAHA* gene in different follicles and changes after FSH treatment

**Figure 5:**
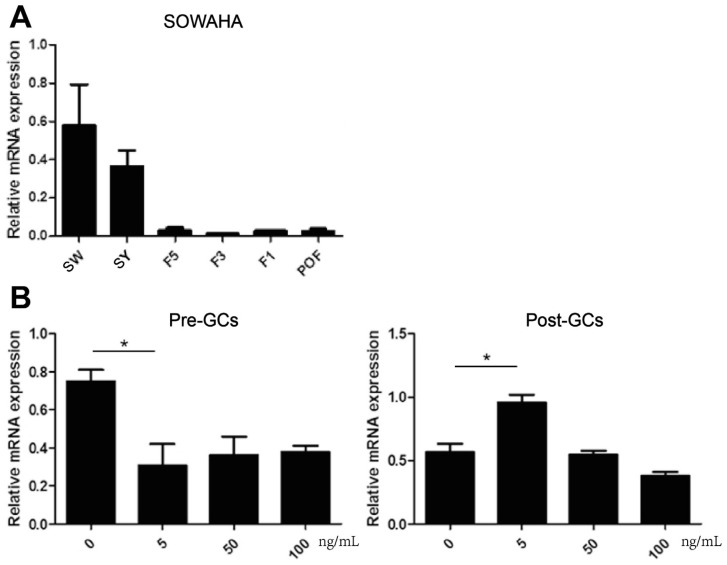
Expression of *SOWAHA* mRNA in different chicken follicles (A) and is affected by FSH treatment in granulosa cells of chicken prehierarchal and hierarchal follicles (B). *SOWAHA*, sosondowah ankyrin repeat domain family member A; FSH, follicle stimulation hormone.

The expression of *SOWAHA* was higher in SW follicles and SY follicles, markedly decreased in hierarchal follicles (F5, F3, and F1), and slightly increased in post-ovulation follicles (POFs) (Figure 5A). After FSH treatment, the mRNA expression of *SOWAHA* was significantly decreased (p<0.05) in granulosa cells of prehierarchal follicles, while it was increased (p<0.05) in hierarchal follicles granulosa cells after treatment with FSH at 5 ng/mL (Figure 5B).

### Effect of *SOWAHA* on follicular development related genes in granulosa cells of prehierarchal and hierarchal follicles

**Figure 6:**
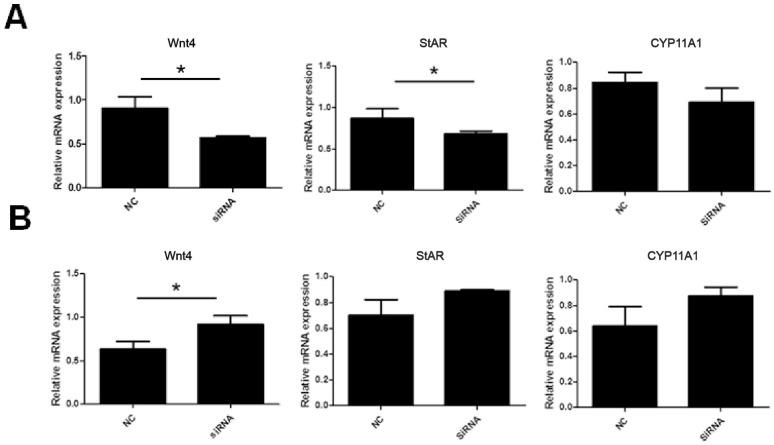
Expression of *Wnt4*, *STAR*, and *CYP11A1* mRNA in the granulosa cells of chicken prehierarchal (A) and hierarchal follicles (B) that is affected by SOWAHA knockdown. *Wnt4*, Wnt family member 4; *StAR*, steroidogenic acute regulatory protein; *CYP11A1*, cytochrome P450 family 11 subfamily A member 1; *SOWAHA*, sosondowah ankyrin repeat domain family member A.

**Figure 7:**
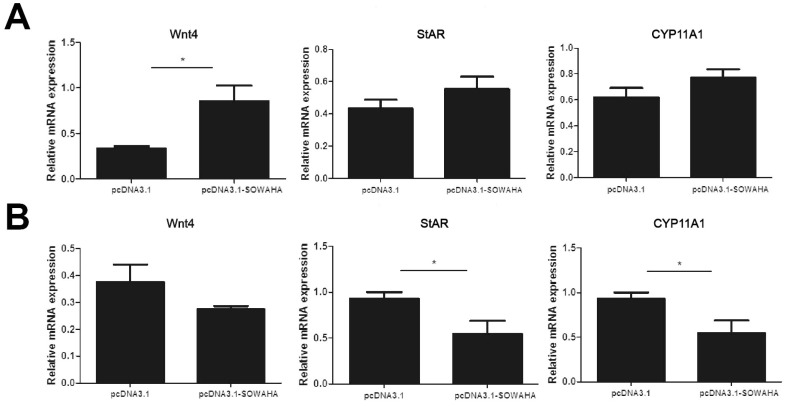
Expression of *Wnt4*, *StAR*, and *CYP11A1* mRNA in the granulosa cells of chicken prehierarchal (A) and hierarchal (B) follicles that is affected by SOWAHA overexpression. *Wnt4*, Wnt family member 4; *StAR*, steroidogenic acute regulatory protein; *CYP11A1*, cytochrome P450 family 11 subfamily A member 1; *SOWAHA*, sosondowah ankyrin repeat domain family member A.

In the granulosa cells of chicken prehierarchal follicles, the expression of of Wnt family member 4 (*Wnt4*) and steroidogenic acute regulatory protein (*StAR*) mRNA was significantly decreased (p<0.05) when *SOWAHA* was knockdown by small interfering RNA (Figure 6A). However, after similar treatment, the expression of *Wnt4* was significantly increased (p<0.05) in the granulosa cells of chicken hierarchal follicles (Figure 6B). After overexpression of chicken *SOWAHA* gene, the expression of *Wnt4* was significantly increased (p<0.05) in prehierarchal follicles granulosa cells (Figure 7A), while the expression of *StAR* and cytochrome P450 family 11 subfamily A member 1 (*CYP11A1*) were significantly decreased (p<0.05) in hierarchal follicles granulosa cells (Figure 7B).

### Effects of *SOWAHA* on the proliferation of chicken granulosa cells

**Figure 8:**
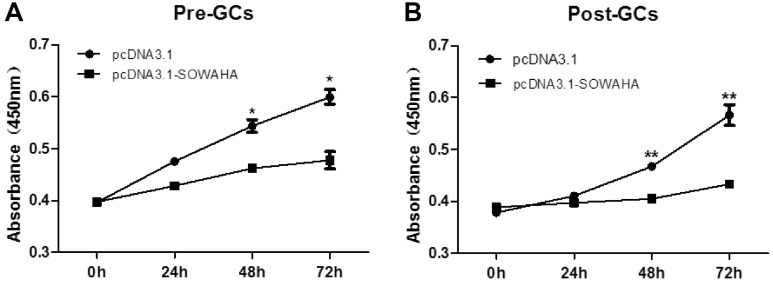
Effect of *SOWAHA* on the proliferation of granulosa cells of both hierarchal and hierarchal follicles in chicken. Overexpression of chicken *SOWAHA* gene significantly inhibited (p<0.05) the proliferation of granulosa cells from both prehierarchal (A) and hierarchal follicles (B). *SOWAHA*, sosondowah ankyrin repeat domain family member A.

Granulosa cells were transfected with the overexpression vector pcDNA3.1-SOWAHA and empty pcDNA3.1 to examine the effect of *SOWAHA* on their proliferation. We found that overexpression of chicken *SOWAHA* gene significantly inhibited (p<0.05) the proliferation of granulosa cells from both prehierarchal (Figure 8A) and hierarchal follicles (Figure 8B).

## DISCUSSION

Systemic transcriptomic analysis on expression differences between chicken SY follicles differing in *FSHR* expression is helpful for elucidating mechanisms underlying follicle selection in chicken. By previous study we uncovered the role of *Wnt4* in chicken follicle selection [4]. In this study, for the first time, we performed an integrated transcriptomic analysis on these SY follicles, identified an interaction network of *SOWAHA*, *miRNA-6615-3p*, *miRNA-1560-3p*, *miRNA-3540*, and *lncRNA-210520.2* and examined the effect of *SOWAHA* on *Wnt4*, *StAR*, and *CYP11A1* mRNA expression, as well as on the proliferation of granulosa cells.

*SOWAHA*, also called ankyrin repeat domain-containing protein 43, is conserved in mammalian species including chimpanzee, Rhesus monkey, dog, cow, mouse, and rat, however, its homology with chicken *SOWAHA* is rather lower (33%). Genomic sequencing indicated that, despite 600 million years of independent evolution, Iroquois genes are linked to ankyrin-repeat-containing *SOWAHA* genes in nearly all studied bilaterians [12]. Three studies investigated the role of *SOWAHA* in mice and human. In HeLa-derived TZM-bl cells, knockdown of *SOWAHA* by siRNA inhibits HIV-1 replication [13]. During development, the expression of murine *SOWAHA* is observed in the postmitotic mantle zone during the specification and patterning of progenitor cells [14] and it is significantly downregulated by the exposure of nonsteroidal anti-inflammatory drugs to the canine melanoma cell line [15]. In this study, we found a dramatic decline of *SOWAHA* in hierarchal follicles and POFs than in prehierachical follicles and FSH treatment brought about different effect in granulosa cells of prehierarchal and hierarchal follicles. In SY follicles, the one with the highest *FSHR* expression would be stimulated by circulating FSH, causing a decline in *SOWAHA* due to the inhibitory role of FSH, and this SY follicle will be selected. In granulosa cells of hierarchal follicles, the effect of FSH on *SOWAHA* was not detected except at low concentration (5 ng/mL).

The expression of *StAR* and *CYP11A1* is prerequisite for progesterone synthesis in the granulosa cells of chicken follicles, indicating the occurrence of granulosa cell differention, therefore, is the hallmark of follicle selection [16]. *Wnt4* plays an important role in chicken follicle selection by stimulating granulosa cell proliferation and steroidogenesis [4]. Knockdown and overexpression of *SOWAHA* produced a different effect on the expression of *Wnt4*, *StAR*, and *CYP11A1* in granulosa cells of prehierarchal and hierarchal follicle. In prehierarchal follicles, *SOWAHA* significantly stimulated the expression of *Wnt4*, but its effect on *CYP11A1* was not significant. In hierarchal follicles, however, *SOWAHA* inhibits the expression of *StAR* and *CYP11A1*. Moreover, *SOWAHA* inhibits the proliferation of granulosa cells of both prehierarcha and hierarchal follicles. These results collectively suggest that, during follicle selection, with the increasing expression of *FSHR*, the function of *SOWAHA* would be inhibited and consequently, the expression of *StAR* and *CYP11A1* and granulosa cell proliferation will increase. *SOWAHA* plays an inhibitory role in chicken follicle selection by affecting granulosa cell proliferation and differentiation.

Integrated analysis indicated that the expression of *SOWAHA* was regulated by three miRNAs, i.e. *miRNA-6615-3p*, *miRNA-1560-3p*, *miRNA-3540*, as well as by one lncRNA (*lncRNA-210520.2*) (Figure 4). The role of *miRNA-6615-3p*, *miRNA-1560-3p*, *miRNA-3540*, and *lncRNA-210520.2* in ovarian follicles was not reported. During follicle selection in chicken, how *SOWAHA* is regulated by *miRNA-6615-3p*, *miRNA-1560-3p*, *miRNA-3540*, and *lncRNA-210520.2* needs to be investigated.

In conclusion, in this study, by integrated analysis on chicken SY follicle transcriptomes, we identified *SOWAHA* as a network gene differentially expressed between SY follicles with marked *FSHR* expression. Further *in vitro* analysis indicated that *SOWAHA* was affected by FSH in granulosa cells of ovarian follicles. *SOWAHA* affected the expression of genes involved in chicken follicle selection and inhibited the proliferation of granulosa cells, suggesting an inhibitory role in chicken follicle selection.

## Figures and Tables

**Table 1:** Primers used in this study

Genes	Accession number (GenBank)	Strand	Sequence (5'-3')	Annealing temperature (°C)
*SOWAHA*	XM_015294072.1	F	CCACAATCCAACACTCCACAGGAC	58
		R	TCAGCAGCTTGTTCAAGGACGAAG	
*AREG*	XM_015276315.1	F	TTGGTGAACGCTGTGGTGAA	60
		R	TGATGCTGGAGAGCAGGACA	
*BLK*	XM_004935895.2	F	AGATGCTGAACGGCTGCTGTTG	60
		R	CGGACAAGGAATAGGCACCTGTG	
*CORIN*	XM_015285490.1	F	CAGAGCGAGCCAAGTGGACAC	60
		R	CCTCTGTGTCCTCCTCGAAGATGG	
*DNASE2B*	XM_015290817.1	F	GACGCCACTGAATTCCACCTTCC	56
		R	TTGCTCAACAGCGCGGTTAAGG	
*CENPO*	NM_001031099.1	F	ACCGTGTCAAGTAACAGCAAGACC	60
		R	CTAGCAATGGAAGAGTGCCAGGTG	
*COL4A3*	XM_015276989.1	F	GGTGACCGAGGAGAACCTGGAG	57
		R	TCCTTGTCTGCCAGCCAGTCC	
*FAM189A2*	XM_424828.5	F	GCAAGAAGCAGGTGAGGAGGTTG	60
		R	GCTGAAGGTGGAAGACTCGTTCTG	
*GNS*	NM_001199559.1	F	CTGTTGCCACTGTTGAGAGGAGAC	60
		R	GGACAGGTAGGATCACTGCCATTG	
*RLN1*	NM_001113200.1	F	GAGTTCATCCGTGCCGTCATCTTC	60
		R	CGAGTTGCTTCTCTGCATCTCAGG	
*Wnt4*	NM_240783.1	F	CCTGTCTTTGGCAAGGTGG	58
		R	CATAGGCAATGTTATCGGAGC	
*StAR*	NM_204686	F	TGCCTGAGCAGCAGGGATTTATCA	58
		R	TGGTTGATGATGGTCTTTGGCAGC	
*CYP11A1*	NM_001001756	F	ACTTCAAGGGACTGAGCTTTGGGT	58
		R	AGTTCTCCAGGATGTGCATGAGGA	
*β-actin*	NM_205518	F	TGGATGATGATATTGCTGC	58
		R	ATCTTCTCCATATCATCCC	
*TCONS_00087329*	TCONS_00087329	F	ACCTGGCTCTTCCTGGAACTGAG	60
		R	GCTTCTCATCTTGCTACGCTCCTC	
*XR_001467762.1*	XR_001467762.1	F	AGTTCAGGAGCCTCCGAGACAC	62
		R	TTCTGCTGACCTGACCATTGCTG	
*NR_131198.1*	NR_131198.1	F	AGGAACCGAGGAGGCTCACAC	60
		R	GCTGTAGTCATCCGCACCTGTAC	
*XR_210520.2*	XR_210520.2	F	GCCTGGCCTTGAATGTCTCCTG	59
		R	GCAGAGCTGTGGACAAGAGGTG	
*XR_001466128.1*	XR_001466128.1	F	CCTTGGAACTTCAGGTGCTACAGC	60
		R	GTCCTTGTTCTGCCTGGAAGAGC	
*XR_001467287.1*	XR_001467287.1	F	ACTGCACTCTGCTATGGTTGGTTG	59
		R	AAGCAGGTGCCTTACACTTATGCC	
*XR_001467288.1*	XR_001467288.1	F	CACGTCATTGGAAGGAGGATGCC	62
		R	GTGCTCTGCTCAGTGTTCGGAAG	
*XR_001469608.1*	XR_001469608.1	F	AGATCGAGTCGGAACTGTCACAGG	60
		R	ACGCTGCTGTATGGACATTCACG	
*XR_001466127.1*	XR_001466127.1	F	CCAGGCAGAACAAGGACAGTCAG	60
		R	CATAAGGCAGCAAGAGGCAGGAAG	
*XR_001467128.1*	XR_001467128.1	F	CCTTGGAACTTCAGGTGCTACAGC	60
		R	GTCCTTGTTCTGCCTGGAAGAGC	
*gga-miR-1560-3p*			GCATCTCTGGACGCGCTCGTTC	60
*gga-miR-6615-5p*			TGGCACTGATGTGTTCTCCACA	60
*gga-miR-3540*			ATGGTGGAAGAACAAGGCCTGC	60
*gga-miR-147*			GTGTGCGGAAATGCTTCTGC	60
*gga-miR-6553-3p*			AACACTACCCTGAGCTGCTCC	60
*gga-miR-130c-5p*			GCCCTTTTTATGTTGTACTACT	60
*gga-miR-1434*			GTGCGTGATGATGGAAAATT	60
*gga-miR-460a-3p*			CACAGCGCATACAATGTGGATT	60
*gga-miR-17-3p*			ACTGCAGTGAAGGCACTTGT	60
*gga-miR-3535*			GGATATGATGACTGATTATCTGAAA	60

*SOWAHA*, sosondowah ankyrin repeat domain family member A; *AREG*, Amphiregulin; *BLK*, BLK proto-oncogene, Src family tyrosine kinase; *CORIN*, corin, serine peptidase; *DNASE2B*, deoxyribonuclease 2 beta; *CENPO*, centromere protein O; *COL4A3*, collagen type IV alpha 3 chain; *FAM189A2*, family with sequence similarity 189 member A2; *GNS*, glucosamine (N-acetyl)-6-sulfatase; *RLN1*, relaxin 1; *Wnt4*, Wnt family member 4; *StAR*, steroidogenic acute regulatory protein; *CYP11A1*, cytochrome P450 family 11 subfamily A member 1.
